# Circular RNA POSTN Promotes Myocardial Infarction-Induced Myocardial Injury and Cardiac Remodeling by Regulating miR-96-5p/BNIP3 Axis

**DOI:** 10.3389/fcell.2020.618574

**Published:** 2021-02-18

**Authors:** Nan Cheng, Ming-Yan Wang, Yuan-Bin Wu, Hui-Min Cui, Shi-Xiong Wei, Bing Liu, Rong Wang

**Affiliations:** Department of Cardiovascular Surgery, PLA General Hospital, Beijing, China

**Keywords:** MI, myocardial injury, cardiac remodeling, circPostn, miR-96-5p

## Abstract

Myocardial infarction (MI) is the most prevalent cardiac disease with high mortality, leading to severe heart injury. Circular RNAs (circRNAs) are a new type of regulatory RNAs and participate in multiple pathological cardiac progressions. However, the role of circRNAs Postn (circPostn) in MI modulation remains unclear. Here, we aimed to explore the effect of circPostn on MI-induced myocardial injury and cardiac remodeling. We identified that the expression of circPostn was elevated in the plasma of MI patients, MI mouse model, and hypoxia and reoxygenation (H/R)-treated human cardiomyocytes. The depletion of circPostn significantly attenuated MI-related myocardium injury and reduced the infarct size in MI mouse model. The circPostn knockdown obviously enhanced left ventricular ejection fraction (LVEF) and left ventricular fraction shortening (LVFS) and inhibited left ventricular anterior wall thickness at diastole (LVAWd) and left ventricular posterior wall thickness at diastole (LVPWd). The depletion of circPostn was able to decrease MI-induced expression of collagen 1α1 and collagen 3α1 in the ventricular tissues of mice. The protein expression of collagen and α-smooth muscle actin (SMA) was up-regulated in MI mice and was inhibited by circPostn knockdown. Meanwhile, the expression of atrial natriuretic peptide (ANP) and brain natriuretic peptide (BNP) was repressed by circPostn depletion in the ventricular tissues of MI mice. Besides, the circPostn depletion attenuated cardiomyocyte apoptosis in mice. Mechanically, circPostn served as a miR-96-5p sponge and miR-96-5p-targeted BNIP3 in human cardiomyocytes, in which circPostn up-regulated BNIP3 expression by targeting miR-96-5p. circPostn promoted H/R-induced cardiomyocyte injury by modulating miR-96-5p/BNIP3 axis. Thus, we conclude that circPostn contributes to MI-induced myocardial injury and cardiac remodeling by regulating miR-96-5p/BNIP3 axis. Our finding provides new insight into the mechanism by which circPostn regulates MI-related cardiac dysfunction. circPostn, miR-96-5p, and BNIP3 are potential targets for the treatment of MI-caused heart injury.

## Introduction

Coronary heart disease is featured by the formation of stable atheromas that induce chronic myocardial ischemia or vulnerable plaques that lead to acute occlusive atherothrombotic complications (Shah et al., 2018). Myocardial infarction (MI) is a destructive menace to human beings, and it impacts a growing amount of people globally, which is not only the vital matter of unexpected cardiac mortality but also the essential process driving heart failure (HF) (Saxena et al., 2016; The Lancet, 2016). The heart is remarkably susceptible to hypoxic or ischemic injury, and a persistent and rapid reduction in coronary artery flow secondary to coronary artery occlusion leads to unchangeable cardiomyocyte damage, resulting in subsequent HF and even immediate cardiac death (Lu et al., 2015). The understanding of the mechanism of MI-induced myocardial injury and cardiac remodeling is critical to the development of treatment and management of MI (Frangogiannis, 2015). However, the advancement in this research field is still limited.

Numerous potential curative candidates for MI-related injury have been identified in recent years, such as non-coding RNAs (ncRNAs) (Garikipati et al., 2015; Khan et al., 2015). Circular RNAs (circRNAs) have been recognized as a novel type of regulatory RNAs with gene regulative functions, but their roles in cardiac injury and repair are not well-illustrated (Chen and Yang, 2015; Altesha et al., 2019). These closed covalent transcripts are produced when the pre-mRNA splicing machine back splices to enter the downstream 5′-splice sites to the upstream 3′-splice sites (Altesha et al., 2019). circRNAs are also developing as potential markers of heart disease (Altesha et al., 2019). circRNAs obtained from the FOXO3 loci have been described to have practical functions in cardiac injury, and the circRNA MICRA is able to predict cardiac dysfunction in human patients (Vausort et al., 2016; Du et al., 2017). Moreover, it has been reported that circular RNA101237 regulates MI-related heart injury by modulating let 7a 5p/IGF2BP3 in cardiomyocytes (Gan et al., 2020). circRNAs Postn (circPostn) has presented regulatory functions in cancer progression by regulating cell proliferation and apoptosis (Long et al., 2020). Importantly, circRNA profiling has identified that circPostn is elevated in MI mice (Garikipati et al., 2019). However, the role of circPostn in MI-induced myocardial injury and cardiac remodeling remains unclear.

Circular RNAs exert their functions by several mechanisms, such as peptide encoding, regulating transcription, and binding with microRNAs (miRNAs) (Aufiero et al., 2019; Zhang L. et al., 2020), in which circRNAs can serve as upstream regulators of miRNAs (Li et al., 2019). miRNAs are small ncRNAs containing 19–22 nucleotides, which are significant to various organic processes by modulating targeted genes (Makeyev and Maniatis, 2008). It has been identified that miRNAs are involved in the regulation of MI (Patane and Patane, 2018). MiR-130 increases acute myocardial infarction (AMI)-induced heart injury by inhibiting peroxisome proliferator-activated receptor gamma (PPAR-γ) (Chu et al., 2018). HOTAIR/miR-126 signaling is negatively associated with the risk of MI (Yu and Chen, 2020). A recent study revealed the protective function of MiR-206 in managing the ischemic injury-caused apoptosis of cardiomyocytes by targeting PTP1B (Yan et al., 2020). Moreover, previous investigations show that miR-96-5p impacts cell proliferation and apoptosis by targeting key genes referring to the progression of cancers (He et al., 2018; Wang S. et al., 2020; Yin et al., 2020). However, the role of miR-96 in MI-related heart injury remains unclear. Meanwhile, BNIP3 is a member of the Bcl-2 family and forms firm homodimerizing groups that surround the external membrane of the mitochondria following cellular pressure, promoting either non-apoptotic or apoptotic cell death (Chinnadurai et al., 2008; Burton and Gibson, 2009). It has been recognized that BNIP3 and BNIP3-mediated programmed death play a critical role in HF, especially during ischemia (Webster et al., 2005). Besides, miR-96-5p is able to target BNIP3/FAK signaling to modulate wound healing (Wu et al., 2019). Nevertheless, the correlation of miR-96-5p and BNIP3 with circPostn in MI-induced myocardial injury and cardiac remodeling is still elusive.

In this study, we aimed to explore the role of circPostn in the modulation of MI. We identified a novel function of circPostn in promoting MI-induced myocardial injury and cardiac remodeling by modulating miR-96-5p/BNIP3 signaling.

## Materials and Methods

### Patients’ Samples

Blood samples were collected from MI patients (*n* = 50) and healthy cases (*n* = 50) and were obtained from the PLA General Hospital. All healthy cases possessed normal test results, and the laboratory and electrocardiographic echocardiography examinations showed no evidence of cerebrovascular or cardiovascular disorder. The patients who were diagnosed with MI underwent percutaneous coronary intervention (PCI). The MI diagnosis was based on the 2017 ESC Guidelines for the management of AMI in patients presenting with ST-segment elevation. At least one of the following was present in AMI patients: symptoms (i.e., persistent chest pain > 30 min) and signs (i.e., 12-lead electrocardiogram, ST-segment elevation > 0.2 mm) consistent with myocardial ischemia, at least a twofold increase in troponin I (TnI). At least one of the following was present in such patients: typical symptoms (dyspnea and fatigue), specific signs (elevated jugular venous pressure, hepatojugular reflux, pulmonary crackles, and peripheral edema), age-specific levels of N-terminal pro-brain natriuretic peptide (NT-proBNP) (ng/L) (age < 50 years: >450 ng/L, age 50–75 years: >900 ng/L, age > 75 years: >1, 800 ng/L), and left ventricular ejection fraction (LVEF) <50%. This study conformed to the experimental guidelines of the World Medical Association and the Ethics Committee of PLA General Hospital. The samples used in this study were under the written approval of the patients and healthy cases. Blood samples were immediately centrifuged to obtain pure plasma. The samples subsequently were stored at −80°C for further investigation. The characteristics of MI patients were shown in Supplementary Table 1.

### MI Mouse Model

To establish the MI mouse model, the C57BL/6 mice (4–6 months old, male, 25–30 g) were intraperitoneally injected with ketamine (120 mg/kg) and xylazine (5 mg/kg body weight) for anesthetization. The left anterior descending coronary artery occlusion/reperfusion (LAD/reperfusion) was performed. Briefly, we placed the LAD on the heart surface by applying an anatomy microscope and ligated the LAD for 30 min, followed by the restoration of blood flow. Sham mice were conducted the surgery without occlusion of LAD. We injected the lentiviral vectors comprising shRNA of circPostn (Genechem, China) (1 × 10^7^ TU/mice) or the lentivirus comprising control shRNA in the mice ventricular cavity, followed by the construction of the MI mouse model. Then, the heart tissues and blood were collected from mice for further analysis. The cardiac injury was assessed by hematoxylin and eosin (H&E) staining. The cardiac function was analyzed by echocardiography measurement with an ultrasound system (Panoview, China) furnished with a 30-MHz phased-array transducer in 3-day post-MI mice. The circPostn shRNA, control shRNA, miR-96-5p mimic, control mimic, miR-96-5p inhibitor, control inhibitor, and the pcDNA3.1-BNIP3 overexpression vector were obtained (GenePharma, China). Animal care and method procedure were authorized by the Animal Ethics Committee of PLA General Hospital.

### Cell Culture and Treatment

The AC16 cell lines (ventricular cardiomyocyte of humans) were obtained from the American Type Tissue Culture Collection. The cells were cultured in Dulbecco’s Modified Eagle’s Medium (DMEM; Solarbio, China) containing 10% fetal bovine serum (Gibco, United States), 0.1 mg/ml streptomycin (Solarbio, China), and 100 units/ml penicillin (Solarbio, China) at a condition of 37°C with 5% CO_2_. The cell injury model was established by hypoxia and reoxygenation (H/R). The cells without H/R served as control. The cells were cultured in a medium without glucose and fetal bovine serum for 6 h at a hypoxia condition of 5% CO_2_ and 0.1% O_2_ and then were reoxygenated in DMEM containing normal glucose and 10% fetal bovine serum for 12 h. The circPostn shRNA, control shRNA, miR-96-5p mimic, control mimic, miR-96-5p inhibitor, control inhibitor, and the pcDNA3.1-BNIP3 overexpression vector were obtained (GenePharma, China). The transfection in the cells was performed by Liposome 3000 (Invitrogen, United States) according to the manufacturer’s instructions.

### CCK-8 Assays

The cell viability of the AC16 cells was analyzed by the CCK-8 assays. About 3 × 10^4^ AC16 cells were put into 96 wells and cultured for 12 h. Then, the cells were used for transfection or treatment. After 0, 12, 24, 48, 72, and 96 h, the cells were added with a CCK-8 solution (KeyGEN Biotech, China) and cultured for another 2 h at 37°C. The ELISA browser was applied to analyze the absorbance at 450 nm (Bio-Tek EL 800, United States).

### Analysis of Cell Apoptosis

The cells were treated with control shRNA or circPostn shRNA for 24 h, subjected to hypoxia and serum starvation for 6 h, and evaluated for apoptosis. Around 2 × 10^5^ cells were placed on six-well plates. Cell apoptosis was analyzed by using the Annexin V-FITC Apoptosis Detection Kit (CST, United States) according to the manufacturer’s instruction. About 2 × 10^5^ collected and washed cells were collected by binding buffer and were dyed at 25°C, followed by flow cytometry analysis.

### Luciferase Reporter Gene Assay

The Dual-Luciferase Reporter Assay System (Promega, United States) was used to carry out the luciferase reporter gene assays. Briefly, the cells were treated with the control mimic or miR-96-5p mimic, the vector containing circPostn, circPostn with the miR-96-5p-binding site mutant, BNIP3, and BNIP3 with the miR-96-5p-binding site mutant fragment, by using Opti-MEMRI (Invitrogen, United States), followed by the analysis of luciferase activities, in which Renilla was applied as a normalized control.

### Quantitative Reverse Transcription-PCR

The total RNAs were extracted by TRIzol (Invitrogen, United States). The first-strand cDNA was used as per the manufacturer’s instruction (ReverTra Ace, Japan). The quantitative reverse transcription-PCR (qRT-PCR) was carried out by applying SYBR Real-time PCR I kit (Roche, Germany). The standard controls for miRNA and mRNA/long ncRNA (lncRNA) were U6 and GAPDH, respectively. Quantitative determination of the RNA levels was conducted in triplicate independent experiments.

### Western Blot Analysis

Total proteins were extracted from the cells or mice heart tissues with RIPA buffer (Beyotime, China). Protein concentrations were measured using the BCA Protein Quantification Kit (YEASEN, China). Identical quantities of protein were divided by SDS-PAGE (12.5% polyacrylamide gels), followed by the transfer to PVDF membranes (Millipore, United States). The membranes were hindered with 5% milk and hatched overnight at 4°C with the primary antibodies for collagen 1 (CST, United States), α-smooth muscle actin (SMA) (CST, United States), atrial natriuretic peptide (ANP) (CST, United States), brain natriuretic peptide (BNP) (CST, United States), Bcl-2 (CST, United States), Bax (CST, United States), caspase-3 (CST, United States), β-actin (1:1,000) (CST, United States), and BNIP3 (CST, United States), in which β-actin served as a control. Then, the corresponding secondary antibodies (CST, United States) were used for hatching the membranes for 1 h, followed by visualization by using an Odyssey CLx Infrared Imaging System. The results of western blot analysis were quantified by ImageJ software.

### Statistical Analysis

Data were expressed as mean ± SD, and the statistical analysis was conducted by GraphPad prism 7. The unpaired Student’s *t*-test was used for comparison of two groups, and the one-way ANOVA was used for comparison among multiple groups. *p* < 0.05 was considered as statistically significant.

## Results

### The Expression of circPostn Is Elevated in the Plasma of MI Patients and MI Models

**FIGURE 1:**
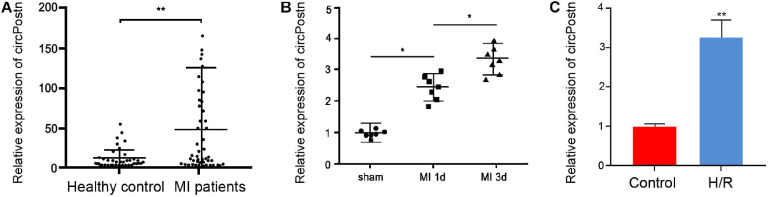
The expression of circPostn is elevated in the plasma of MI patients and MI models. **(A)** The expression levels of circPostn were measured by qPCR in plasma from MI patients (*n* = 50) and healthy controls (*n* = 50). **(B)** The expression levels of circPostn were analyzed by qPCR in the heart tissues of 1- and 3-day post-MI and sham mice (*n* = 6). **(C)** The expression levels of circPostn were assessed by qPCR in the H/R-treated AC16 cells. Data are presented as mean ± SD. Statistically significant differences were indicated: *p < 0.05, **p < 0.01.

To understand the potential correlation of circPostn with MI, we detected the expression of circPostn in clinical MI patients, MI mouse model, as well as H/R-treated cell model. We observed that the expression levels of circPostn were significantly elevated in the plasma of MI patients (*n* = 50) compared with that of healthy controls (*n* = 50) (Figure 1A). Similarly, the expression levels of MI were remarkably elevated in the infarcted myocardial tissues from 1- and 3-day post-MI mice relative to the sham controls (Figure 1B). H/R treatment also enhanced the expression of circPostn in the AC16 cells (Figure 1C). These data imply that circPostn may be closely associated with MI.

### The Depletion of circPostn Relieves Myocardial Injury in MI Mice

**FIGURE 2:**
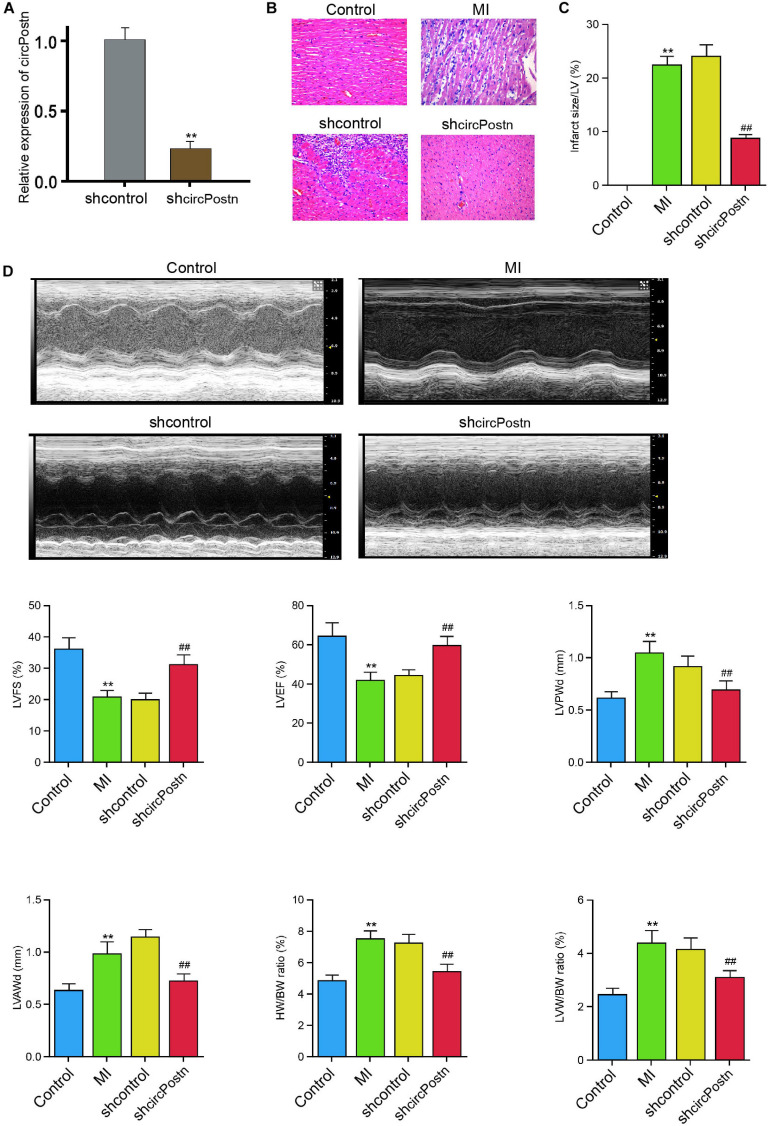
The depletion of circPostn relieves myocardial injury in MI mice. **(A–D)** The left ventricular chamber of 3-day post-MI mice was injected with the lentiviral vector of control shRNA or circPostn shRNA. **(A)** The expression of circPostn was measured by qPCR in mice. **(B)** The myocardium injury was analyzed by hematoxylin and eosin (H&E) staining. **(C)** The infarct size was assessed. **(D)** The LVEF, LVFS, LVAWd, and LVPWd were measured by echocardiography analysis. The ratio of heart weight (HW)/body weight (BW) and left ventricle weight (LVW)/BW was calculated. Data are presented as mean ± SD. Statistically significant differences were indicated: **p < 0.01, ^##^*p* < 0.01.

To identify the effect of circPostn on MI-induced myocardial injury and cardiac remodeling *in vivo*, we injected the lentiviral vector of circPostn shRNA in the mice ventricular chamber and constructed the MI mouse model for 3 days, and the efficiency of circPostn knockdown was validated (Figure 2A). H&E staining showed that the depletion of circPostn significantly attenuated MI-related myocardium injuries (Figure 2B) and reduced the infarct size (Figure 2C). Echocardiography analysis revealed that MI decreased LVEF and left ventricular fraction shortening (LVFS) and increased left ventricular anterior wall thickness at diastole (LVAWd) and left ventricular posterior wall thickness at diastole (LVPWd), whereas the depletion of circPostn could obviously enhance LVEF and LVFS and inhibited LVAWd and LVPWd (Figure 2D). Besides, circPostn knockdown remarkably reduced the ratio of heart weight (HW)/body weight (BW) and left ventricle weight (LVW)/BW (Figure 2D). Together, these data suggest that the depletion of circPostn relieves myocardial injury in MI rats.

### The Depletion of circPostn Attenuates Cardiac Remodeling in MI Mice

**FIGURE 3:**
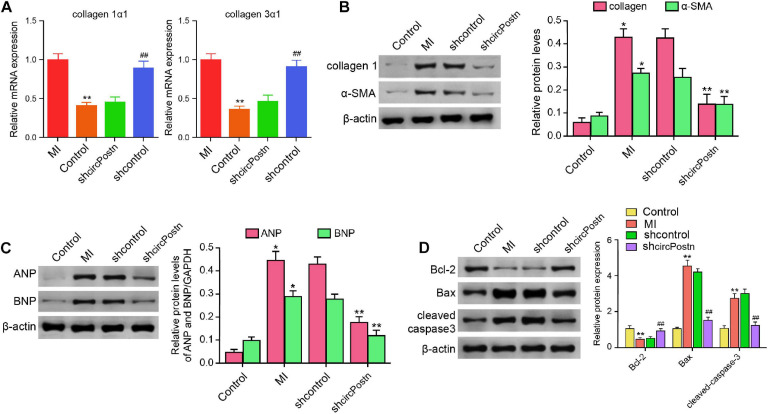
The depletion of circPostn attenuates cardiac remodeling in MI mice (Rzem et al., 2007). The left ventricular chamber of 3-day post-MI mice was injected with the lentiviral vector of control shRNA or circPostn shRNA. **(A)** The mRNA expression of collagen 1α1 and collagen 3α1 was measured by qPCR in mice. **(B)** The protein expression of collagen and α-SMA was tested by western blot analysis in mice. The results of western blot analysis were quantified by ImageJ software. **(C)** The protein expression of ANP and BNP was assessed by western blot analysis in mice. The results of western blot analysis were quantified by ImageJ software. **(D)** The protein expression of Bcl-2, Bax, cleaved caspase-3, and β-actin was assessed by western blot analysis in mice. The results of western blot analysis were quantified by ImageJ software. Data are presented as mean ± SD. Statistically significant differences were indicated: *p < 0.05, **p < 0.01, ^##^*p* < 0.01.

Next, we were interested in the role of circPostn in MI-related cardiac remodeling *in vivo.* Given that previous studies have identified that collagen, α-SMA, ANP, and BMP serve as the markers of myocardial fibrosis and remodeling (Wang J. et al., 2020; Zhang B. F. et al., 2020), we further evaluated the effect of circPostn on these factors. The depletion of circPostn was able to reduce the MI-induced expression of collagen 1α1 and collagen 3α1 in the ventricular tissues (Figure 3A). Meanwhile, the protein expression of collagen and α-SMA was up-regulated in MI mice, in which circPostn knockdown could reduce their expression in MI mice (Figure 3B). Moreover, the expression of ANP and BNP was inhibited by the circPostn depletion in the ventricular tissues of MI mice (Figure 3C). In addition, the anti-apoptotic Bcl-2 expression was reduced, but the pro-apoptotic Bax and cleaved caspase-3 expression was enhanced in MI mice, in which the depletion of circPostn could reverse this phenotype (Figure 3D). Together, these data suggest that the depletion of circPostn attenuates cardiac remodeling in MI mice.

### circPostn Serves as a miR-96-5p Sponge in Human Cardiomyocytes

**FIGURE 4:**
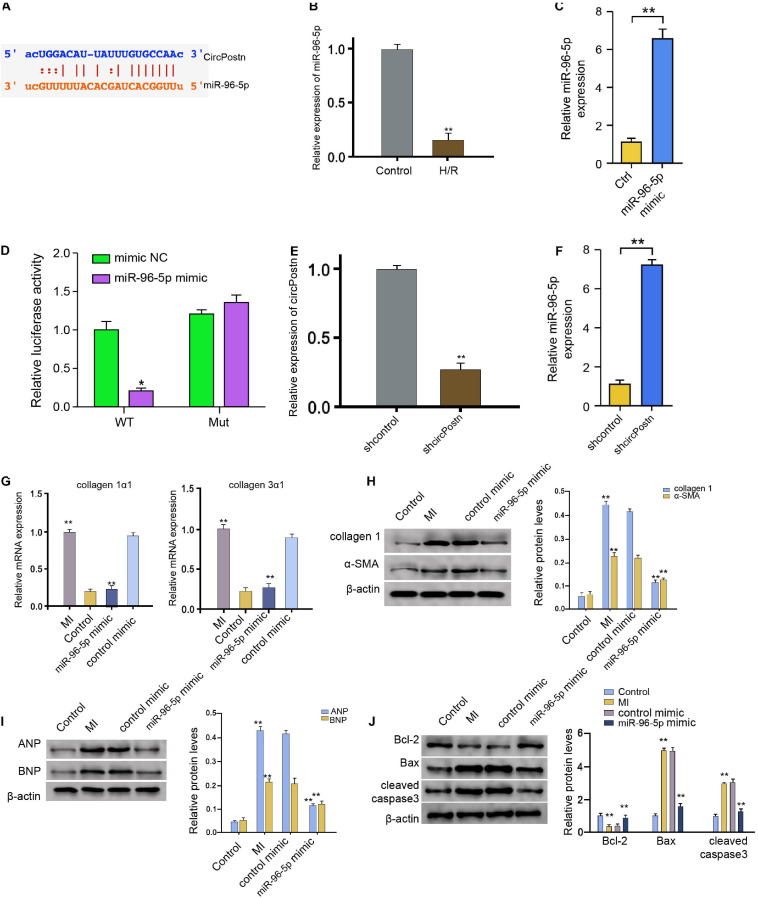
circPostn serves as a miR-96-5p sponge in human cardiomyocytes. **(A)** The potential interaction between circPostn and miR-96-5p was identified in a bioinformatic analysis by using ENCORI (see text footnote 1). **(B)** The expression levels of miR-96-5p were assessed by qPCR in the H/R-treated AC16 cells. **(C)** The expression levels of miR-96-5p were measured by qPCR in the AC16 cells treated with control mimic or miR-96-5p mimic. **(D)** The luciferase activities of wild-type circPostn (circPostn WT) and circPostn with the miR-96-5p-binding site mutant (circPostn MUT) were determined by luciferase reporter gene assays in the AC16 cells treated with control mimic or miR-96-5p mimic. **(E,F)** The AC16 cells treated with control shRNA or circPostn shRNA. **(E)** The expression of circPostn was measured by qPCR in mice. **(F)** The expression of miR-96-5p was measured by qPCR in the AC16 cells. **(G–J)** The left ventricular chamber of 3-day post-MI mice was injected with the control mimic or miR-96-5p mimic. **(G)** The mRNA expression of collagen 1α1 and collagen 3α1 was measured by qPCR in mice. **(H)** The protein expression of collagen and α-SMA was tested by western blot analysis in mice. The results of western blot analysis were quantified by ImageJ software. **(I)** The protein expression of ANP and BNP was assessed by western blot analysis in mice. The results of western blot analysis were quantified by ImageJ software. **(J)** The protein expression of Bcl-2, Bax, cleaved caspase-3, and β-actin was assessed by western blot analysis in mice. The results of western blot analysis were quantified by ImageJ software. Data are presented as mean ± SD. Statistically significant differences were indicated: *p < 0.05, **p < 0.01.

We then further explored the underlying mechanism of circPostn-mediated myocardial injury and cardiac remodeling induced by MI. We identified a potential interaction between circPostn and miR-96-5p in a bioinformatic analysis by using ENCORI^1^ (Figure 4A). H/R treatment also reduced the expression of miR-96-5p in the AC16 cells (Figure 4B). We treated the AC16 cells with control mimic or miR-96-5p mimic, and the efficiency was verified in the cells (Figure 4C). The miR-96-5p mimic remarkably reduced the luciferase activities of wild-type circPostn but failed to affect the circPostn with the miR-96-5p-binding site mutant (Figure 4D). Then, the cells were treated with control shRNA or circPostn shRNA, and the efficiency of circPostn knockdown was validated (Figure 4E). Moreover, the expression of miR-96-5p was reduced by the depletion of circPostn in the cells (Figure 4F). The miR-96-5p mimic was able to reduce the MI-induced expression of collagen 1α1 and collagen 3α1 in the ventricular tissues (Figure 4G). Meanwhile, the protein expression of collagen and α-SMA was up-regulated in MI mice, in which miR-96-5p mimic could reduce their expression in MI mice (Figure 4H). Moreover, the expression of ANP and BNP was inhibited by the miR-96-5p mimic in the ventricular tissues of MI mice (Figure 4I). In addition, the anti-apoptotic Bcl-2 expression was reduced, but the pro-apoptotic Bax and cleaved caspase-3 expression was enhanced in MI mice, in which miR-96-5p mimic could reverse this phenotype (Figure 4J). Together, these data indicate that circPostn serves as a miR-96-5p sponge in human cardiomyocytes.

### MiR-96-5p Targets BNIP3 in Human Cardiomyocytes

**FIGURE 5:**
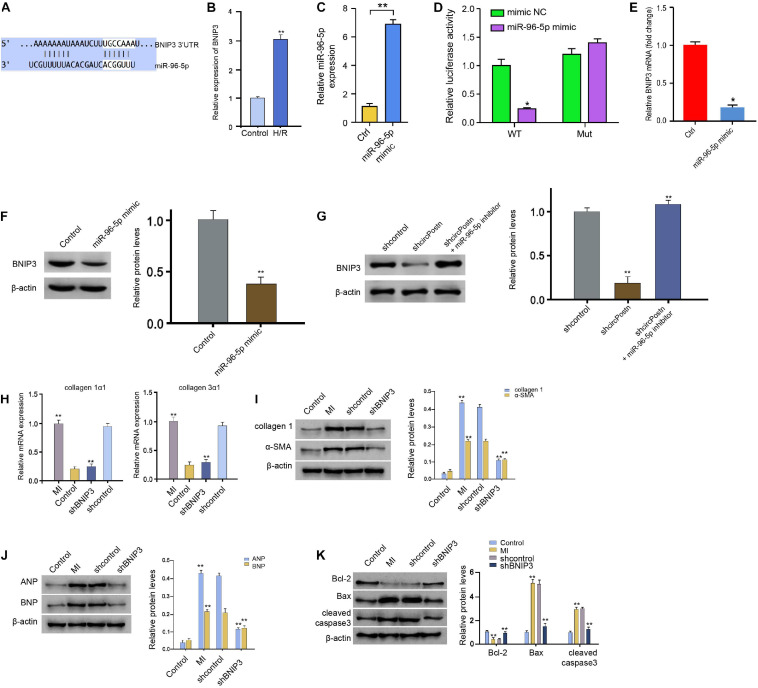
MiR-96-5p targets BNIP3 in human cardiomyocytes. **(A)** The interaction of BNIP3 and miR-96-5p was identified by bioinformatic analysis using Targetscan (see text footnote 2). **(B)** The expression levels of BNIP3 were assessed by qPCR in the H/R-treated AC16 cells. **(C)** The expression levels of miR-96-5p were measured by qPCR in the AC16 cells treated with control mimic or miR-96-5p mimic. **(D)** The luciferase activities of wild-type BNIP3 (BNIP3 WT) and BNIP3 with the mutant of the miR-96-5p-binding site (BNIP3 MUT) were analyzed by luciferase reporter gene assays in the AC16 cells treated with control mimic or miR-96-5p mimic. **(E)** The mRNA expression of BNIP3 was measured by qPCR in the AC16 cells treated with control mimic or miR-96-5p mimic. **(F)** The protein expression of BNIP3 was tested by western blot analysis in the AC16 cells treated with control mimic or miR-96-5p mimic. The results of western blot analysis were quantified by ImageJ software. **(G)** The protein expression of BNIP3 was analyzed by western blot analysis in the AC16 cells treated with control shRNA, circPostn shRNA, or co-treated with circPostn shRNA and miR-96-5p inhibitor. The results of western blot analysis were quantified by ImageJ software. **(H–K)** The left ventricular chamber of 3-day post-MI mice was injected with the lentiviral vector of control shRNA or BNIP3 shRNA. **(H)** The mRNA expression of collagen 1α1 and collagen 3α1 was measured by qPCR in mice. **(I)** The protein expression of collagen and α-SMA was tested by western blot analysis in mice. The results of western blot analysis were quantified by ImageJ software. **(J)** The protein expression of ANP and BNP was assessed by western blot analysis in mice. The results of western blot analysis were quantified by ImageJ software. **(K)** The protein expression of Bcl-2, Bax, cleaved caspase-3, and β-actin was assessed by western blot analysis in mice. The results of western blot analysis were quantified by ImageJ software. Mean ± SD of at least three experiments was shown. Statistically significant differences were indicated: *p < 0.05, **p < 0.01.

Next, we identified the miR-96-5p-targeted site in BNIP3 3′ UTR in a bioinformatic analysis by using Targetscan^2^ (Figure 5A). H/R treatment also enhanced the expression of circPostn in the AC16 cells (Figure 5B). We treated the AC16 cells with control mimic or miR-96-5p mimic, and the efficiency was verified in the cells (Figure 5C). Remarkably, the miR-96-5p mimic inhibited the luciferase activities of wild-type BNIP3 but failed to affect the BNIP3 with the miR-96-5p-binding site mutant in the AC16 cells (Figure 5D). The mRNA expression of BNIP3 was attenuated by miR-96-5p mimic in the cells (Figure 5E), suggesting that miR-96-5p targets BNIP3. A similar result was also observed in the analysis of the protein expression of BNIP3 (Figure 5F). Moreover, the expression levels of BNIP3 were attenuated by circPostn knockdown, in which miR-96-5p inhibitor rescued this effect (Figure 5G), indicating that circPostn up-regulates BNIP3 expression by targeting miR-96-5p. Furthermore, the depletion of BNIP3 was able to reduce the MI-induced expression of collagen 1α1 and collagen 3α1 in the ventricular tissues (Figure 5H). Meanwhile, the protein expression of collagen and α-SMA was up-regulated in MI mice, in which BNIP3 knockdown could reduce their expression in MI mice (Figure 5I). Moreover, the expression of ANP and BNP was inhibited by the BNIP3 depletion in the ventricular tissues of MI mice (Figure 5J). In addition, the anti-apoptotic Bcl-2 expression was reduced, but the pro-apoptotic Bax and cleaved caspase-3 expression was enhanced in MI mice, in which the depletion of BNIP3 could reverse this phenotype (Figure 5K).

### circPostn Promotes H/R-Induced Cardiomyocyte Injury by Modulating miR-96-5p/BNIP3 Axis

**FIGURE 6:**
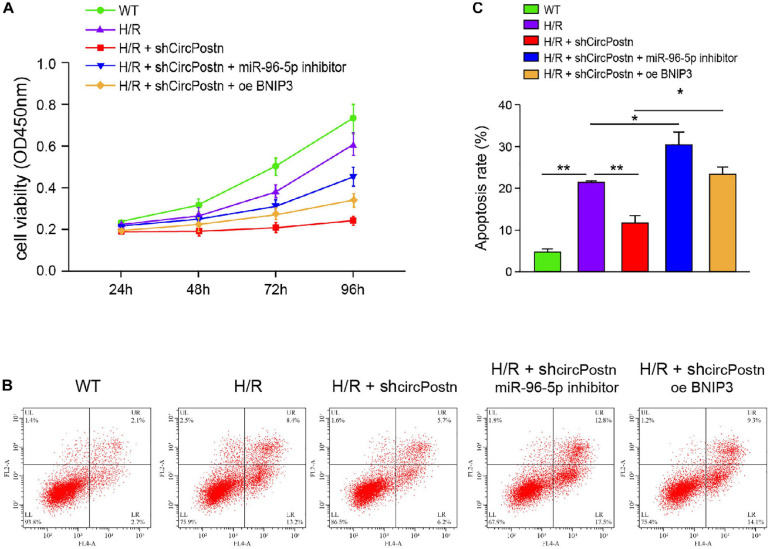
circPostn promotes H/R-induced cardiomyocyte injury by modulating miR-96-5p/BNIP3 axis (Rzem et al., 2007). The H/R-treated AC16 cells were treated with circPostn shRNA or co-treated with circPostn shRNA and miR-96-5p inhibitor or BNIP3 overexpression vector. **(A)** The cell viability was measured by CCK-8 assays in the cells. **(B,C)** Cell apoptosis was analyzed by flow cytometry in the cells. Mean ± SD of at least three experiments was shown. Statistically significant differences were indicated: *p < 0.05, **p < 0.01.

We further determined the role of circPostn/miR-96-5p/BNIP3 signaling in H/R-induced injuries of cardiomyocytes. Notably, the depletion of circPostn enhanced cell viability, in which the miR-96 inhibitor or the BNIP3 overexpression blocked the cell viability in the H/R-treated AC16 cells (Figure 6A). Meanwhile, the H/R treatment enhanced the apoptosis of AC16 cells, and the depletion of circPostn inhibited H/R treatment-induced apoptosis, in which miR-96 inhibitor and BNIP3 overexpression could reverse the effect of circPostn depletion in the system (Figures 6B,C). Together, these data suggest that circPostn promotes H/R-induced cardiomyocyte injury by modulating miR-96-5p/BNIP3 axis.

## Discussion

Myocardial infarction is one of the most severe cardiovascular disorders (Lim, 2017; Williams et al., 2018). MI is a principal problem for mortality and morbidity globally, which is described by pathological alterations correlated with hypoxia and myocardial ischemia (Lim, 2017; Williams et al., 2018). In the clinical setting, MI is regularly due to a coronary vessel thrombotic occlusion induced by the vulnerable plaque rupture (Karlsson et al., 2019). Ischemic injury and cardiac remodeling are the significant complication and harmful insults of MI patients, containing substantial ionic and metabolic disturbances in the injured myocardium and causing rapid systolic function depression (Alpert and Thygesen, 2016; Montaigne et al., 2018). Meanwhile, it has been identified that circRNAs play crucial roles in the development of MI. Nevertheless, the effect of circPostn on MI-induced myocardial injury and cardiac remodeling remains elusive. In the present study, we identified that circPostn promoted MI-induced myocardial injury and cardiac remodeling by regulating miR-96-5p/BNIP3 axis.

As a critical modulator in multiple pathological processes, circRNAs are found to participate in the modulation of MI-induced myocardial injury and cardiac remodeling. It has been reported that circRNA ACR inhibits myocardial injury by repressing autophagy through regulating the Pink1/FAM65B signaling (Zhou et al., 2019). circRNA Cdr1as increases MI by modulating miR-7a and its target expression (Geng et al., 2016). circRNA CircFndc3b regulates MI-induced cardiac injury by FUS/VEGF-A signaling (Garikipati et al., 2019). circRNA Ttc3 mediates cardiac function after MI by targeting miR-15b (Cai et al., 2019). Circ_0068655 enhances cardiomyocyte apoptosis by miR-498/PAWR signaling (Chai et al., 2020). In this study, we first identified that the expression of circPostn was elevated in the plasma of MI patients and MI rat models. The depletion of circPostn relieved myocardial injury and cardiac remodeling in MI rats. These data present a novel function of circPostn in MI-related cardiac dysfunction, providing valuable evidence for the fundamental role of circRNAs in the development of heart disease.

As another critical member of ncRNA and the primary interaction factors with lncRNAs in the physiological and pathological settings, miRNAs are also involved in the modulation networks of MI as well as MI-induced heart injury. It has been reported that the nanoparticle transfer of miRNA-21 to heart macrophages ameliorates cardiac remodeling after MI (Bejerano et al., 2018). The circulating miR-320a is a portentous biomarker for remodeling of ventricular in MI cases experiencing original percutaneous coronary interference (Galeano-Otero et al., 2020). The curative potential of miR-9b and miR-19a in defending heart function by systemically remitting miR-9b and miR-19a into post-MI mice has been identified (Gao et al., 2019). In addition, it has been reported that miR-96 is a critical regulator in heart disease. MiR-96-5p participates in angiotensin II-mediated cardiac fibroblasts, and the repression of miR-96 can inhibit cardiac fibrosis by increasing KLF13 (Su et al., 2020). The miR-96-5p expression is inhibited in patients with cardiac cardiomyopathy and may serve as a biomarker for heart fibrosis (Szemraj-Rogucka et al., 2019). Furthermore, it has been reported that targeting BNIP3 in inflammation-related cardiac failure is a practical therapeutic strategy for heart disease (Fordjour et al., 2016). BNIP3 depletion contributes to the inhibition of ischemia cell apoptosis in post-MI mice (Diwan et al., 2007). Our mechanism investigation uncovered that miR-96-5p was sponged by circPostn and miR-96-5p could target BNIP3 in human cardiomyocytes. These data present an unreported role of miR-96-5p in MI progression, identifying the new upstream circPostn and downstream BNIP3 mRNA of miR-96 in response to MI-related heart injury.

## Conclusion

In conclusion, we discovered that circPostn contributed to MI-induced myocardial injury and cardiac remodeling by regulating miR-96-5p/BNIP3 axis. Our finding provides new insight into the mechanism by which circPostn regulates MI-related cardiac dysfunction. circPostn, miR-96-5p, and BNIP3 are potential targets for the treatment of MI-caused heart injury.

## Data Availability Statement

The original contributions presented in the study are included in the article/Supplementary Material, further inquiries can be directed to the corresponding author/s.

## Ethics Statement

The studies involving human participants were reviewed and approved by the PLA General Hospital. The ethics committee waived the requirement of written informed consent for participation.

## Author Contributions

RW and NC: data curation. RW: formal analysis. NC: funding acquisition and investigation. M-YW: methodology, project administration, and resources. Y-BW and BL: software. Y-BW: supervision. H-MC: validation and writing—original draft. S-XW: visualization. S-XW and BL: writing—review and editing. All authors contributed to the article and approved the submitted version.

## Conflict of Interest

The authors declare that the research was conducted in the absence of any commercial or financial relationships that could be construed as a potential conflict of interest.

## Footnotes

1 http://starbase.sysu.edu.cn/index.php

2 http://www.targetscan.org/vert_72/
