# Supplementary material for: Circular RNA POSTN Promotes Myocardial Infarction-Induced Myocardial Injury and Cardiac Remodeling by Regulating miR-96-5p/BNIP3 Axis
Source: Front Cell Dev Biol. 2021 Feb 18;8:618574. doi: 10.3389/fcell.2020.618574 (PMC7930329; doi:10.3389/fcell.2020.618574)
Supplement: Supplementary file 1 [file Table_1.DOCX]

**Supplementary Table 1: The characteristics of MI patients**

| **Variable** | **Value** |
| --- | --- |
| Age (years) | 58.3 ± 8.5 |
| Diabetes, % | 25% |
| Hypertension, % | 51% |
| Hyperlipidemia, % | 75% |
| Prior myocardial infarction, % | 27% |
| Body mass index (kg/m^2^) | 28.3 ± 5.5 |
| Systolic blood pressure (mmHg) | 129 ± 18 |
| Diastolic blood pressure (mmHg) | 85 ± 10 |
| ST-elevation myocardial infarction, % | 43% |
| Aspirin use | 82% |
| Beta blocker use | 85% |
| Angiotensin converting enzyme inhibitor use | 65% |
| Serum total cholesterol, mg/dl | 125 ± 63 |
| Serum high density lipoprotein, mg/dl | 28 ± 22 |
| Serum low density lipoprotein, mg/dl | 85 ± 45 |
| Serum triglycerides, mg/dl | 105 ± 90 |
